# Testing the ABCs of Serious Illness Program for Oncology Trainees: A Feasibility Trial Comparing Different Learning Formats for a Virtual Communication Curriculum

**DOI:** 10.1177/26892820251376359

**Published:** 2025-09-15

**Authors:** Oren Levine, Jeff Myers, Shilpa Jyothi Kumar, Daryl Bainbridge, Leah Steinberg, Nadia Incardona, Ghazaleh Kazemi, Hsien Seow

**Affiliations:** ^1^ Department of Oncology, McMaster University, Hamilton, Canada.; ^2^ Department of Family and Community Medicine, University of Toronto, Toronto, Canada.

**Keywords:** communication, medical education, oncology, palliative care, serious illness conversation, virtual training

## Abstract

**Background:**

ABCs (All providers, Better Communication skills) of serious illness communication is a novel curriculum that could enhance postgraduate oncology training. The program combines electronic learning modules (ELMs), standardized patient (SP) encounters, and coaching, all in a virtual format. We assessed feasibility of a randomized controlled trial (RCT) comparing different training experiences.

**Methods:**

We conducted a pilot for a RCT at three academic centers. Postgraduate oncology trainees were randomized to complete ELMs and SP encounters with coaching (intervention arm) versus ELMs only (control arm). Feasibility measures comprised the primary analysis. Secondary analyses explored the impact of different training experiences. Outcomes were measured through pre- and post-intervention simulation-based assessments (COM-ON rating scale), and surveys rating self-efficacy (End-of-Life Professional Caregiver Survey [EPCS] score) and satisfaction.

**Results:**

Twenty-three learners participated (37% recruitment). Adherence was 100% and data collection was near complete. All feasibility metrics were met except for the recruitment target of 75%. Self-efficacy ratings improved from baseline (EPCS score increased significantly (*p* < 0.001), mean paired difference = 0.73 (standard deviation [SD] = 0.78) [95% confidence interval (CI), 0.37–1.08]), as did quality of communication in simulations (COM-ON score increased significantly (*p* < 0.001), mean paired difference = 0.59 (SD = 0.73) [95% CI, 0.28–0.91]). Improvement was greatest in the intervention arm for both. Participants reported high satisfaction with virtual learning.

**Conclusions:**

Recruitment was below target, but study activities were feasible in virtual format. The curriculum improved communication skills. The addition of virtual SPs and coaching optimized learning. The curriculum was associated with improved self-efficacy rating for serious illness communication with oncology patients. Lessons learned will support further medical education research in this area.

## Background 

Cancer is a devastating illness for many patients. Difficult conversations are common in oncology practice and patient-centered communication is essential to care for individuals with cancer.^1^ High quality communication is associated with benefits that include improved quality of life and satisfaction among patients, improved bereavement outcomes for families, improved satisfaction and reduced burnout for clinicians.^2–5^ Residency training is an opportunity to develop the necessary skills for independent practice including communication skills. Proficiency in a variety of communication tasks is now a requirement within competency-based medical education (CBME) in Canada.^6,7^ Within oncology residency programs, however, communication training is mostly unstructured observation and feedback in the clinic and many learners receive inadequate training.^8^ Currently, educational resources are limited, and residents have indicated a desire for more training on end-of-life and serious illness communication skills.^9^ A formal communication curriculum could fill a gap and help to standardize teaching and evaluation.

Several training programs that address serious illness communication skills for health care professionals have been initiated over the past decade, including the Serious Illness Care Program, VitalTalk, and Speak Up.^10–12^ Limitations of these programs are that they are narrow in focus, e.g., specific to advanced care planning and goals of care, are oriented to treatments, or do not cover the full spectrum of difficult conversations between diagnosis of a serious illness and end-of-life care.^13–17^ There are also considerable inconsistencies and variability among serious illness communication training in defined purpose, terminology, essential components, and expected outcomes, with there being no standard format for building these important competencies among medical residents. Virtual platforms for structured teaching remain commonplace in many training programs in the post-pandemic era, with large scale potential yet how best to teach communication skills in the context of virtual clinical and educational interactions is unclear.^17^ We adapted two recognized educational tools, electronic learning modules (ELMs) and standardized patients (SPs), to create a virtual training strategy. ELMs allow for delivery of didactic teaching with flexibility on the timing and pace of accessing content, while overcoming geographic barriers to in-person learning. The value of ELMs has been shown for oncology trainees for teaching topics ranging from the basic science of chemotherapy to communication of advanced directives with cancer patients.^18,19^ Moreover, with internal medicine learners, communication training for code status discussions was equally effective through an ELM compared to a live workshop.^19^ ELMs could be leveraged to support communication skills training on a broader range of topics.

The ABCs (All providers, Better Communication skills) is a virtual education program that builds skills and person-centered strategies for listening to, talking with and supporting seriously ill patients and family. This education integrates SP simulated encounters and coaching to actively facilitate competency development. SPs can create a high fidelity simulated patient encounter and improve self-reported comfort in communication skills such as delivery of bad news.^20,21^ Typically, SP skills sessions occur in person and effectiveness of SP encounters in a virtual care context has not been evaluated. Moreover, it is uncertain whether SPs are necessary for improvement in transfer of skills for end-of-life communication training. Thus, we aim to evaluate the relative impact of each component of a virtual communication curriculum including ELMs and SPs. In this study, we explored the feasibility of a randomized controlled trial of the ABCs education comparing different virtual learning experiences for serious illness communication training in oncology residency programs.

## Methods

### Design

This is a randomized controlled trial (RCT) feasibility study of an educational intervention involving a virtual communication skills curriculum for oncology residents. Ethics approval for this study was received from the Hamilton Integrated Research Ethics Board (HIREB) (#14948). We followed the SPIRIT reporting guidelines.^22^

### ABCs education intervention

**FIG. 1.**
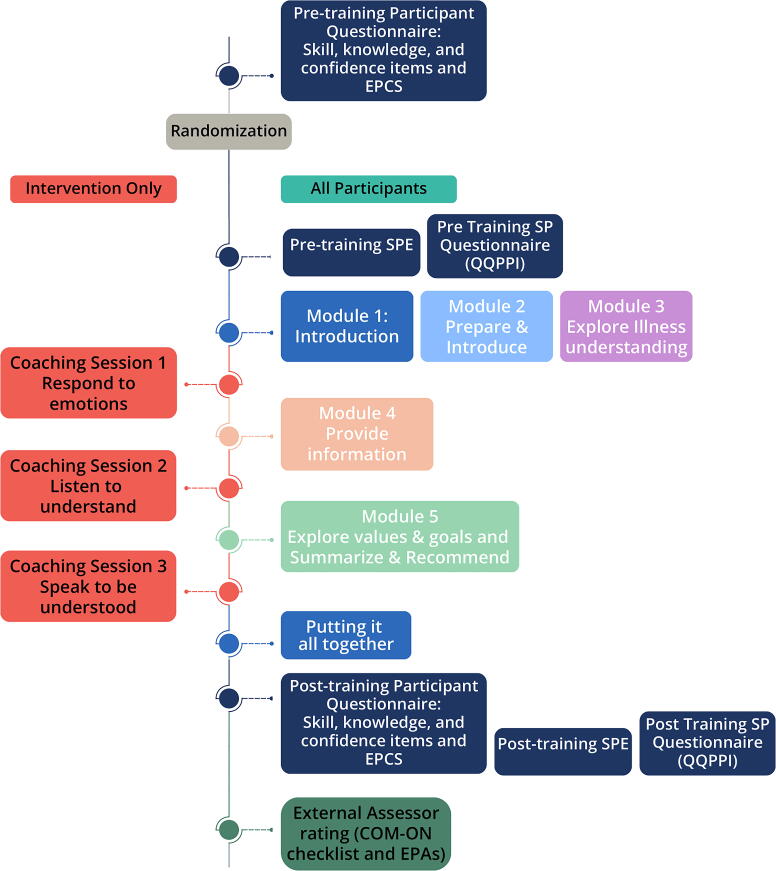
ABCs Program Timeline Depicting Pre and Post Data Collection and Assessments, Core Educational Components, and Intervention Components Across Study Arms. Study design and data collection timeline. All participants completed six virtual modules and participated in both pre- and post-intervention questionnaires as well as a SPE. SPs also completed pre- and post-questionnaires evaluating their interactions with participants. Only participants randomized to the intervention arm received additional coaching sessions, each of which included an SPE followed by feedback from a clinical coach. All pre- and post-intervention SPEs were recorded, and interactions were independently rated after data collection was complete by external assessors. COM-ON, communication in Oncology checklist; EPAs, Entrustable Professional Activities scale; EPCS, End-of-Life Professional Caregiver Survey; SPE, simulated patient encounter; SPs, simulated patients; QQPPI, quality of physician-patient interaction.

Researchers and clinicians at the University of Toronto and McMaster University developed the ABCs (All providers, Better Communication Skills) program, an ELM-based virtual curriculum that teaches core communication skills for health care professionals, which underlie a wide range of conversations about serious illness (Fig. 1). This virtual education was developed to teach communication building blocks that can be transferable to different types of conversations with seriously ill patients and their families, as previously described in detail.^17^ The ABCs content was informed by over two decades of providing communication workshops in palliative care (J.M., N.I., and L.S.) and a review of existing relevant programs to avoid duplication.^23–29^

The education intervention in this study was the online content from the ABCs program (Oncology version). The curriculum consists of six 30 (to 40)-minute virtual modules comprised of video-cases, educational text, and short quizzes, over 12 weeks in total. The modules cover the topics of: (i) introduction to conversations about serious illness; (ii) preparing for and introducing a conversation; (iii) assessing understanding; (iv) providing information; (v) responding to emotion; and (vi) summarizing and recommending a plan. Within each module, there were micro-skills—simple and actionable techniques to show residents how they can apply communication best practices.

### Setting/subjects

Medical and radiation oncology residents and fellows at McMaster University, and medical oncology residents at the Universities of Ottawa and Toronto, all in Ontario, Canada, were invited to participate. Inclusion criteria were enrollment in a participating training program, ability to participate in study-related activities in English, and access to Internet and a video-enabled computer (either personal or institutional) to support study-related activities. The proposed sample size was 40 (20 per arm), based on achieving a 95% confidence interval (CI) of 60% to 85% around an estimated recruitment proportion of 75%. We deemed this sample size feasible for this pilot study. Recruitment occurred through email invitations distributed by program administrators and further mention by program directors of this training opportunity. Recruitment occurred at 2 time points spanning 2 academic years to achieve sample size. Based on limited recruitment at the first time point, the protocol was amended to offer a $75 gift card as incentive for participation. The program was delivered in two separate cohorts.

### Study groups and randomization

Residents that consented to participate in the study were randomized 1:1 into one of two groups using a computer-generated randomization sequence (D.B.). Allocation concealment was achieved through a centralized computer-generated group assignment. Participants in the experimental arm received access to the online electronic learning modules (ELMs) and 3 virtual SP encounters with coaching feedback, while those in the control arm only received the ELMs. The ELMs were hosted on a Moodle learning management system. Upon commencement of the program, one ELM was released every two weeks (via Moodle) for learners to complete. At time points staggered through the curriculum, participants in the intervention arm individually completed virtual SP encounters (over Zoom video conferencing platform) that allowed the participant to apply the skills taught in the prior ELMs. Scenarios involving patients with advanced cancer diagnoses were developed using case templates from the McMaster Center for Simulation Based Learning. A faculty preceptor either from medical oncology, or palliative care, was present to provide coaching feedback. Participants also received feedback from the SP regarding communication style. The virtual SP encounter and feedback was capped at 30 minutes. These sessions were booked according to learner availability to avoid conflicting with other scheduled curricular activities.

### Data collection

Participants completed online surveys on Moodle before and two weeks after the ABCs program to assess satisfaction with the learning experience and self-efficacy rating in conducting conversations about serious illness with cancer patients (Fig. 1). Participants in both study groups also completed a virtual simulated patient encounter, pre and post intervention, that was recorded. The clinical scenarios (pre = lung cancer, post = prostate cancer) were provided to participants in advance and an experienced SP simulated the encounter (Supplementary Fig. S1). The scenarios were reviewed with the SPs for clarity, comprehensiveness and authenticity, to ensure high psychological fidelity. The SPs completed a patient-directed survey immediately following each simulated encounter, rating the quality of physician communication. Once all simulated encounters had concluded, the video recordings were randomly assigned to trained assessors, who asynchronously reviewed and scored each video on the communication skills of the learner. Assessors were oriented to the rating scale through a virtual meeting and were provided with scoring manuals. Assessors were not from the same institution as the learner to reduce bias and to protect anonymity. The study investigators, SPs, and assessors of the recording were blind to participant allocation and encounter timing (e.g., pre or post ABCs). SurveyMonkey was used to collect SP and assessor responses.

### Outcomes

The primary outcome was whether our feasibility metrics, such as recruitment, randomization, representativeness, adherence to intervention, and completeness of data collection (See Table 1) met the criteria for success. Secondary outcomes related to participant competency in communication assessed through validated measures pre and post intervention. Participant self-rating was captured through surveys. SPs and external assessors provided ratings of participant communication in virtual simulated patient encounters.

**Table 1.** Feasibility Metrics and Criteria for Success

Metric	Threshold for success	Achieved
Recruitment and randomization (proportion of eligible residents enrolling on study and consenting to randomization)	75%	36.5% (23 of 63 potential participants)
Representativeness (sample characteristics including site, program, year of training)	All sites, oncology disciplines, and both junior/senior residents are represented in the sample	Diversity of residency years and oncology disciplines, participants predominately from one site.
Adherence to intervention (participants completing all modules and simulated SP encounters according to assigned group)	70%	100%
Satisfaction (with curricular format and virtual learning experience)	70% of participants providing an overall favorable satisfaction rating with the virtual learning experience on a Likert scale	66.7% strongly agreed and 33.3% agreed that overall, the program was effective in helping them develop communication skills for clinical practice
Rate of participation in both ELM curriculum and video-recorded assessments	80%	100%
Completeness of data collection	<10% missing data from surveys and OSCE scores	No missing data except from 2 participants that did not complete the post survey, therefore <10% for post survey

#### Resident participants

The participant pre and post questionnaire included three study created items to assess self-reported skill, knowledge, and confidence in discussing serious illness with patients and families. Items were rated on a five-point Likert scale ranging from 1=“None at all” to 5=“A great deal.” Two open text items in the pre questionnaire asked about their learning goals and the aspects of communication they wished to strengthen.

The questionnaire also included four items from the Patient and Family-Centered Communication sub-domain on the End-of-Life Professional Caregiver Survey (EPCS).^30^ The EPCS is a validated instrument assessing educational needs relating to palliative care for interprofessional members of the health care team. Items are rated on a five-point Likert scale ranging from 1=“lowest level of skill” to 5=“greatest level of skill,” with higher scores denoting a higher level of skill.

Finally, the post intervention version of the questionnaire contained 10 items to assess participant satisfaction with and perceived utility of the training curriculum and format, rated on a five-point Likert scale ranging from 1=‘‘I do not agree’’ to 5=‘‘I fully agree,’’ with higher scores denoting higher satisfaction/usefulness. This post survey also contained open text items for participants to explain why they enrolled in the ABCs and to what extent their learning objectives were met.

#### Standardized patients

SPs completed the Questionnaire on the Quality of Physician-Patient Interaction (QQPPI), a patient-facing rating scale on the quality of physician communication. The 13 items are rated on a five-point Likert scale ranging from 1=‘‘I do not agree’’ to 5=‘‘I fully agree’’, with higher scores denoting higher quality communication.^31^

#### External assessors

Assessors completed the Communication in Oncology (COM-ON) checklist for assessing general and specific communication skills in oncology. The 12 items are rated on a five-point Likert scale ranging from 1=‘‘Skill not demonstrated at all’’ to 5=‘‘Skill fully demonstrated’’, with higher scores denoting higher quality communication.^32^

Assessors also completed medical interaction scale for entrustable professional activities (EPAs) based on a rubric defined by the Royal College of Physicians and Surgeons of Canada, specific to the communication tasks in each scenario: breaking bad news (8 items) and transitioning to end of life care (9 items).^7^ Items are rated on a three-point scale as to whether the particular milestone was: 1=“Not observed”, 2=“In Progress”, or 3 =“Achieved”.

In addition, both the SPs and the external assessors provided a global rating of the quality of the learner’s communication in each simulated encounter (same scale as COM-ON).

### Data analysis

Standard descriptive statistics (frequencies, mean, median, standard deviation [SD], etc.) were calculated for participant characteristics and the outcomes. Inferential (paired t-tests) statistical analyses with corresponding 95% confidence intervals (CIs) were conducted to compare pre to post intervention individual paired differences overall. The significance level was set at α = 0.05 for all statistical tests, and analyses were conducted using two-tailed tests. Analyses were completed using IBM SPSS version 29. Our research team conducted a thematic analysis of the open text responses.

## Results

We conducted the pilot between February 2023 and May 2024. We recruited 23 medical and radiation oncology residents and fellows, all who completed the study. Nearly all (*n* = 19) trainees were from McMaster University, with few recruited from the other universities (3 = University of Toronto, 1 = University of Ottawa). About 40% (*n* = 9) of the residents were in fourth year, with the rest distributed between first and seventh years of postgraduate training. The majority (60.9%) were women, and the mean age overall was 31.8 years (range 28 to 42 years). 12 residents were allocated to the intervention arm and 11 residents to the control arm.

### Feasibility

Our feasibility metrics for this study and our findings are presented in Table 1. The greatest challenge to feasibility was accruing residents to the study, with less than half (37%) of those approached agreeing to participate in the training/study. Among those accrued, retention was absolute, with all 23 residents fully completing the education program. Overall satisfaction with the program was high, with all participants agreeing or strongly agreeing that the program was effective in helping them develop communication skills for clinical practice.

### Resident participant satisfaction with education

Participant satisfaction with various aspects of the ABCs was high, ranging from a mean of 4.3 to 4.8 (on five-point scale where higher scores equal stronger agreement) (Table 2). There was no discernable difference between study groups in satisfaction ratings.

**Table 2.** Resident Satisfaction Responses (n = 21 Unless Specified)

Satisfaction item	Mean^a^	Standard deviation
The electronic learning format was enjoyable	4.33	0.66
The electronic learning format was clear to understand	4.62	0.50
The content of the communication curriculum was useful	4.67	0.48
I will be able to apply the content of this curriculum in my clinical work	4.67	0.48
I would recommend this curriculum to other oncology trainees	4.67	0.58
Overall, this communication curriculum was effective in helping me develop communication skills for clinical practice	4.67	0.48
Intervention (coaching sessions with virtual standardized patient encounters) group only (n = 10)		
The virtual standardized patient encounters were a helpful training exercise	4.70	0.48
The virtual standardized patient encounters provided an opportunity to practice my communication skills in a nonthreatening environment	4.60	0.52
I received adequate feedback regarding my communication skills in the virtual standardized patient encounters	4.80	0.42

^a^ Scored from 1 = ‘‘I do not agree’’ to 5 = ‘‘I fully agree.’’

### Study group and pre/post intervention comparisons

#### Resident participant Self-Report

All resident self-reported efficacy and EPCS items improved following the education from baseline (pre) for both study groups (Table 3). Overall, the summary mean EPCS score increased significantly (*p* < 0.001), mean paired difference = 0.73 (SD = 0.78) [95% CI, 0.37–1.08]), indicating that the residents reported greater comfort in engaging in these conversations following the ABCs. Much greater improvements in self-efficacy and EPCS items were seen in the intervention group, compared to the control group, across all items (EPCS overall mean paired difference = 0.93 intervention, 0.55 control) (significance not tested).

**Table 3.** Resident Participant Responses (n = 23)

	Pre		Post		Intervention group (N = 12)		Control group (N = 11)	
	Mean	SD	Mean	SD	Mean difference*	SD	Mean difference^a^	SD
Self efficacy								
Thinking about discussing serious illness with patients and families, how much skill do you have?	1.91	0.79	2.86	0.57	1.10	0.74	0.73	0.79
Thinking about discussing serious illness with patients and families, how much knowledge do you have?	2.04	0.71	3.05	0.74	1.30	0.48	0.82	0.60
Thinking about discussing serious illness with patients and families, how much confidence do you have?	1.87	0.81	2.71	0.85	1.10	0.99	0.64	0.50
EPCS items								
I am comfortable helping patients and families to accept a poor prognosis	2.35	0.83	2.95	0.74	0.70	0.95	0.45	0.82
I am able to set goals of care with patients and families	2.39	0.72	3.24	0.54	0.90	0.74	0.82	0.98
I am comfortable starting and participating in discussions about code status	2.78	0.67	3.24	0.62	0.80	1.03	0.27	0.47
I encourage patients and families to complete advance care planning	2.00	1.21	3.05	0.74	1.30	1.34	0.64	1.12
EPCS overall mean	2.38	0.73	3.12	0.55	0.93	0.85	0.55	0.71

^a^ Paired Mean Post–Mean Pre by group.

Higher scores indicate higher agreement (5-point scale).

EPCS, End-of-Life Professional Caregiver Survey; SD, standard deviation.

#### Standardized patients

Overall, SPs ratings of the simulated encounters improved for most but not all (*n* = 5) of the QQPPI items, following the intervention (from baseline) (Supplementary Table S1). Overall, no significant change (*p* = 0.41) in the summary mean QQPPI score occurred, mean paired difference = 0.13 (SD = 0.75) [95% CI, −0.19–0.45]). Little difference was seen between the study groups on this outcome (QQPPI overall mean paired difference = 0.12 intervention, 0.14 control) (significance not tested).

#### External assessors

All COM-ON items evaluated by the external assessors, from the recorded videos of the simulated encounters, improved following the education from baseline (pre) for both study groups (Supplementary Table S2). Overall, the summary mean COM-ON score increased significantly (*p* < 0.001), mean paired difference = 0.59 (SD = 0.73) [95% CI, 0.28–0.91]), indicating that the residents objectively exhibited greater competency in managing these conversations following the ABCs. Much greater improvements in these items were seen in the intervention group, compared to the control group, across all items (COM-ON overall mean paired difference = 0.84 intervention, 0.32 control) (significance not tested). External assessors’ global ratings of conversations showed a similar trend in improvement to the COM-ON summary mean, overall mean paired difference = 0.65 (SD = 0.79, *p* < 0.001) [95% CI, 0.31–0.99]), and study group differences (Global rating overall mean paired difference = 0.96 intervention, 0.32 control, significance not tested).

The individual items of the Pre (lung cancer) and Post (prostate cancer) scenario EPAs were different and therefore not comparable (Supplementary Table S3). Summarizing these scores, the overall mean paired difference of the EPAs = 0.37 (SD = 0.44, *p* < 0.001) [95% CI, 0.17–0.56]), representing improvement from pre to post ABCs. Greater improvements in the EPA summary mean were seen in the intervention group, compared to the control group (EPA overall mean paired difference = 0.53 intervention, 0.19 control) (significance not tested).

### Resident participant qualitative responses

All 21 residents that completed the final survey provided responses to the open text questions. The main reasons cited for enrolling in the ABCs were that participants wanted to build confidence and improve communication skills, particularly around breaking bad news, active listening, discussing poor prognoses, advance care planning and fostering meaningful patient discussions and rapport without compromising trust. Participants expressed goals focused on learning techniques for effective and empathetic communication, particularly for serious illness discussions and patient engagement. The residents expressed that they appreciated the structured approach to serious illness communication and the ABCs improved their confidence in discussing serious illness topics like understand patients values and exploring understanding, provide information in a delicate and empathetic way and providing recommendations based on information gathered.

## Discussion

We tested a novel communication skills training program, the ABCs program (Oncology version) delivered in a virtual format with ELMs, and participants in the intervention group also completed virtual SP sessions receiving individualized coaching. The overall goal of this curriculum is to help trainees in Oncology to build skill as serious illness communicators which is important for high quality care.

Through this pilot study, we explored the feasibility of a randomized controlled trial comparing different training experiences. All the feasibility metrics we measured were met, except for the set recruitment target of 75% (37% obtained). Adherence to and satisfaction with educational activities was very high and data collection was near complete. Thus, the main barrier to successfully conducting a larger RCT is recruitment. Although this is an identified area of need according to oncology learners,^9^ and a variety of engagement strategies were employed (endorsement from program director, testimonials from learners, local champions at each site, gift card incentive), recruitment was well below target and disproportionately representative of one center. This may reflect the challenge of offering optional extra-curricular learning opportunities for trainees that already have busy schedules with academic and clinical activities. The duration of the study and necessary time commitment may have also been barriers. In future, alternative study designs could be considered framed as program evaluation in which learning activities are mandatory and protected time is provided within the workday. Namely, a cluster randomized controlled trial, allocated by site, where the study curriculum is a required training experience, would likely be most successful at including the population of learners.

In exploratory pre/post education analyses, we found that both residents’ self-perceived competency and comfort in engaging in these conversations and external assessors’ ratings of the simulated encounters increased significantly following residents’ completion of the ABCs. While significant improvement in communication skill occurred in both study groups (online modules only versus online modules and coaching) for these outcomes, greater advancement was apparent in the intervention group. Although we designed the ELMs with interactive aspects (e.g., activity where learners categorize prompts according to communication domains, such as responding to emotion, with real-time feedback) to the extent this modality allows, our findings suggest that the SP encounters with individual coaching added value to the training experience. As part of active learning, the education literature has cited skill practice and formative feedback to be integral parts of the learning process and knowledge uptake.^33,34^ Simulation-based learning involves two types of authenticity which contribute to the learning experience: engineering/structural (the physical characteristics of the encounter) and psychological/functional fidelity (the clinical task).^35,36^ The psychological fidelity of an encounter has the greatest impact on improving transfer of skill.^37^ This supports a potential role for SPs even in a virtual format which may lack engineering fidelity. Our results suggest that indeed SP encounters in virtual format enhanced communication skills training in this group of learners. While this is hypothesis generating, a larger study is needed to confirm findings.

A recent scoping review of education modalities for serious illness communication training found that few studies report clinician behavior changes, impact on patient outcomes, or long-term clinician skill acquisition.^17^ Existing literature does not clarify the impact of different modalities for developing competence in serious illness communication skills due to the heterogeneity in education approaches. The scoping review highlights the need for well-designed education intervention studies the can define the value of different education modalities in transfer of skill. Our findings support the potential benefit of SPs and coaching in addition to an ELM curriculum. In future, rigorous evaluations including patient-level outcomes are needed to establish the impact on quality of care.

Our study has several strengths. The ABCs course was well received by participants and offers a suite of specific communication skills that can be adapted to any serious illness encounter. This novel educational resource is delivered in virtual format which is significant for several reasons. It simulates virtual care, a new paradigm that has persisted post-pandemic. Virtual educational formats have also persisted since they allow more flexibility for off-site learners and preceptors to participate. For small programs or universities unaffiliated with SP programs, the remote access to virtual SPs offers a promising new approach. Finally, in this pilot we have gained important experience with the conduct of all study related activities by fully electronic means (dissemination of resources, scheduling and completing all educational activities, and data collection).

The major limitation of this feasibility trial was low accrual and the study not meeting the estimated sample size. Thus, comparisons between groups are exploratory. Yet, the differences observed between study groups is encouraging and we see value in further study of the current curriculum. Additional lessons were learned regarding study execution. We chose to use different clinical scenarios for the pre (lung cancer), and post (prostate cancer) education standardized patient simulated encounters, with the post scenario deemed to be more challenging for participants. This difference created issues in compatibility between pre and post outcomes, particularly evident with participants scoring worse on some standardized patient rated items (QQPPI), post intervention. In addition, the EPA items for the external assessors were different for each scenario and therefore items were not individually comparable pre/post. Although the EPA rubric directly reflects evaluation criteria for resident advancement in training, a standardized and validated rating tool specific to serious illness communication would be of most value to further educational research in this field. A further limitation in representativeness is that residents that agreed to participate may have been more inclined to favor and benefit from ELMs, introducing a volunteer bias. In this multi-site trial, more than 80% of trainees were recruited from the principal investigator’s institution, emphasizing the challenges of engaging residents in education not integrated into the core curriculum. Finally, in this pilot study we did not assess the sustainability of the communication improvements observed, and whether the active learning components contribute to learning retention, a consideration for future research.

## Conclusion

Our pilot RCT of the ABCs program (Oncology version) demonstrated feasibility and a trend potential impact in improving residents’ self-efficacy and expert assessed competency in communication with seriously ill cancer patients. We can reasonably conclude that the SP encounters and coaching were key contributors to the observed improvements in residents’ communication in this trial. Experience to date is encouraging with successful execution of the virtual learning activities, positive experiences reported from participants, and a signal towards enhanced communication skills through this course. Outcomes of this pilot will allow us to refine processes (e.g., recruitment strategy and outcome measures) and plan for a larger study to establish the impact of the ABCs curriculum and different training experiences for oncology learners. In future, this work will be adapted to other medical fields and inter-professional health care team members.

## Ethics Approval and Consent to Participate

Ethical approval for this study was obtained from the Hamilton Integrated Research Ethics Board (#14948). Informed consent to participate was obtained from all subjects involved in the study.

## Consent for Publication

This article contains no personally identifiable information on study participants.

## Supplementary Material

Supplementary Data

Supplementary Figure S1

Supplementary Tables

## Data Availability

De-identified data are available from the corresponding author upon reasonable request.

## Abbreviations Used

ABCs: All providers, Better Communication skills
CBME: Competency-Based Medical Education
COM-ON: Communication in Oncology
ELMs: Electronic Learning Modules
EPAs: Entrustable Professional Activities
EPCS: End-of-Life Professional Caregiver Survey
RCT: Randomized Controlled Trial
SD: Standard Deviation
SP: Standardized Patient
QQPPI: Questionnaire on the Quality of Physician-Patient Interaction
