# Supplementary material for: Testing the ABCs of Serious Illness Program for Oncology Trainees: A Feasibility Trial Comparing Different Learning Formats for a Virtual Communication Curriculum
Source: Palliat Med Rep. 2025 Sep 15;6(1):436–45. doi: 10.1177/26892820251376359 (PMC12543435; doi:10.1177/26892820251376359)
Supplement: Supplementary Figure S1 [file 26892820251376359_supp_figures1.docx]

**Supplemental Figure 1. Clinical Scenarios for Pre and Post Simulated Patient Encounters**

**Pre-training Simulated Patient Encounter Case Scenario summary for clinician:**

Elaine McCallum is a 67-year-old woman you have followed in the thoracic oncology clinic. She has a diagnosis of stage IIIB non-small cell lung cancer. She completed definitive chemoradiation with platinum doublet chemotherapy. She was intending to undergo consolidation immunotherapy.

Past medical history includes HTN and COPD. She is a retired realtor. She is married and has 2 adult children. She has a 40 pack-year smoking history and quit 5 years ago.

Following completion of chemoradiation, she was admitted to hospital with cough and dyspnea. A CTPE did not show any thrombosis, but there were new lesions seen in the sternum and ribs. Bone scan confirms new diffuse bone metastasis. She has been discharged from hospital after stabilization of dyspnea (with prednisone and puffers). She is booked for a virtual assessment with the oncology clinic to review her test results. She was told about ‘lesions’ and to see her oncology team for more information. In future, she will be a candidate for palliative intent systemic treatment and possibly radiation if there are painful bone metastases.

*The purpose of this encounter is to share the test results with Elaine, and to explain the implication of finding metastatic disease. You have 15 minutes for this encounter.*

**Post-training Simulated Patient Encounter Case Scenario summary for clinician**:

Georgina Turnbull is a 60-year-old woman you have followed for a diagnosis of metastatic breast cancer (ER+, Her2 low). She has liver, nodal, and extensive bone metastasis. She has undergone numerous lines of systemic treatment (endocrine therapies, chemotherapy, antibody-drug conjugate). She has received palliative radiation for painful bone metastases.

Past medical history includes HTN and dyslipidemia. She is a retired bank manager. She is married and has 3 adult children.

HPI: She has been struggling with more bone pain (worst areas are the pelvis and ribs). She takes Hydromorph Contin, dexamethasone and routine Tylenol, but is requiring more breakthrough dosing of analgesia. Her appetite is poor, and she has been losing weight.

She is able to get around the house with support from her spouse but spends most of the day resting or napping on the couch or in bed. ECOG =3.

Her Ca 15-3 tumor marker level has been rising rapidly in the last few months despite treatments. She had a bone scan that shows progressive metastasis with new lesions in bilateral ribs, spine and pelvic bones. She had a CT that also shows marked progression in the liver with diffuse metastases. She is booked for a virtual encounter to review the scan results and to discuss next steps.

Based on poor functional status, there are no further conventional therapies to offer, and she is unfit for clinical trials. She is likely in the last 3 months of life.

*The purpose of this encounter is to share the test results with Georgina, and to discuss transitioning away from active cancer treatment. You have 15 minutes for this encounter.*
