# Supplementary material for: Testing the ABCs of Serious Illness Program for Oncology Trainees: A Feasibility Trial Comparing Different Learning Formats for a Virtual Communication Curriculum
Source: Palliat Med Rep. 2025 Sep 15;6(1):436–45. doi: 10.1177/26892820251376359 (PMC12543435; doi:10.1177/26892820251376359)
Supplement: Supplementary Tables [file 26892820251376359_supp_tables.docx]

**Supplemental Table 1. Standardized Patient Responses – QQPPI (n=23)**

| **QQPPI Items** | **Pre** | | **Post** | | **Intervention group (N=12)** | | **Control group (N=11)** | |
| --- | --- | --- | --- | --- | --- | --- | --- | --- |
|  | **Mean** | **SD** | **Mean** | **SD** | **Mean difference*** | **SD** | **Mean difference*** | **SD** |
| Seemed to be genuinely interested in my problems | 4.57 | 0.73 | 4.35 | 0.65 | -0.42 | 1.08 | 0.00 | 0.77 |
| Gave me detailed information about the available treatment options | 4.05 | 0.86 | 3.82 | 0.59 | -0.45 | 0.69 | 0.00 | 1.25 |
| I felt I could have trusted with my private problems | 4.35 | 0.71 | 4.22 | 0.85 | -0.25 | 1.06 | 0.00 | 0.89 |
| Made all treatment decisions together | 3.77 | 0.69 | 4.00 | 0.71 | 0.30 | 1.06 | 0.20 | 1.03 |
| Explanations were easy to understand | 4.17 | 0.83 | 4.09 | 0.73 | -0.08 | 1.00 | -0.09 | 0.83 |
| Spent sufficient time on my consultation | 4.52 | 0.59 | 4.43 | 0.51 | 0.00 | 0.85 | -0.18 | 0.60 |
| Spoke to me in detail about the risks and side effects of the proposed treatment | 3.52 | 1.21 | 3.85 | 0.80 | -0.60 | 1.14 | -0.17 | 1.17 |
| Understood my needs and problems and took them seriously | 4.26 | 0.75 | 4.61 | 0.50 | 0.50 | 1.00 | 0.18 | 0.40 |
| Did all he/she could to put me at ease | 3.91 | 0.85 | 4.09 | 0.73 | 0.33 | 1.30 | 0.00 | 0.89 |
| Asked about how my illness affects my every day life | 3.17 | 1.19 | 3.95 | 0.79 | 0.91 | 1.70 | 0.73 | 0.90 |
| Gave me enough time to talk about all my problems | 4.17 | 0.78 | 4.48 | 0.51 | 0.42 | 1.08 | 0.18 | 0.75 |
| Respects the fact that I may have a different opinion regarding treatment | 4.05 | 0.49 | 4.22 | 0.74 | 0.09 | 1.14 | 0.36 | 0.50 |
| Gave me detailed information about my illness | 4.17 | 0.94 | 4.22 | 0.60 | -0.08 | 1.00 | 0.18 | 1.17 |
| QQPPI overall mean | 4.06 | 0.62 | 4.19 | 0.46 | 0.12 | 0.88 | 0.14 | 0.61 |

*Paired Mean Post - Mean Pre by group

SD = Standard Deviation, QQPPI = Questionnaire on the Quality of Physician-Patient Interaction, Higher scores indicate higher agreement (5-point scale)

**Supplemental Table 2. External Assessor Responses – COM-ON (n=23)**

| **COM-ON Items** | **Pre** | | **Post** | | **Intervention group (N=12)** | | **Control group (N=11)** | |
| --- | --- | --- | --- | --- | --- | --- | --- | --- |
|  | **Mean** | **SD** | **Mean** | **SD** | **Mean difference*** | **SD** | **Mean difference*** | **SD** |
| Initiating a Conversation: Does the clinician initiate the conversation appropriately? | 3.30 | 0.63 | 3.63 | 0.69 | 0.29 | 0.94 | 0.36 | 0.55 |
| Patient's Perception: Does the clinician manage to get an idea of the patient's perception at the beginning of, or during the consultation? | 3.26 | 0.84 | 3.72 | 0.94 | 0.75 | 0.99 | 0.14 | 0.87 |
| Structure of Conversation: Does the clinician actively give structure to the conversation (set an agenda of central topics) | 2.93 | 0.59 | 3.28 | 0.69 | 0.46 | 0.62 | 0.23 | 0.41 |
| Structure of Conversation: Does the clinician set sub-sections during the course of conversation (in detail)? | 2.35 | 0.55 | 2.83 | 0.79 | 0.54 | 0.78 | 0.41 | 0.49 |
| Patient's Emotions: Does the clinician recognize the patient's emotions and do they name them | 2.74 | 0.94 | 3.39 | 1.04 | 1.08 | 1.24 | 0.18 | 0.98 |
| Patient's Emotions: Does the clinician offer emotional support | 2.76 | 0.94 | 3.46 | 0.99 | 1.04 | 1.23 | 0.32 | 0.75 |
| End of Conversation: Does the clinician summarize the content of the consultation and do they close the conversation appropriately? | 2.85 | 0.79 | 3.39 | 0.88 | 0.71 | 1.03 | 0.36 | 0.81 |
| Communication Skills: Does the clinician use clear and appropriate words during the conversation? | 2.72 | 0.96 | 3.76 | 0.93 | 1.46 | 1.12 | 0.59 | 0.86 |
| Communication Skills: Does the clinician use appropriate non-verbal communication during the consultation? | 2.93 | 0.76 | 3.70 | 0.84 | 1.13 | 0.96 | 0.36 | 0.84 |
| Communication SkillsDoes the clinician adjust their pace during the consultation and do they make appropriate pauses? | 2.98 | 0.92 | 3.61 | 0.77 | 1.00 | 1.17 | 0.23 | 1.03 |
| Communication Skills: Does the clinician offer the patient the chance to ask questions during the consultation? | 3.20 | 0.88 | 3.76 | 0.81 | 0.96 | 0.96 | 0.14 | 0.84 |
| Communication Skills: Does the clinician check whether the patient has understood the consultation? | 2.59 | 0.90 | 3.17 | 0.90 | 0.67 | 0.98 | 0.50 | 0.45 |
| COM-ON overall mean | 2.88 | 0.68 | 3.47 | 0.73 | 0.84 | 0.84 | 0.32 | 0.48 |
| Global Rating: How do you assess the communication skills of the clinician in this conversation? | 3.13 | 0.74 | 3.78 | 0.82 | 0.96 | 0.84 | 0.32 | 0.60 |

*Paired Mean Post - Mean Pre by group

SD = Standard Deviation, COM-ON = Communication in Oncology checklist , Higher scores indicate higher agreement (5-point scale)

**Supplemental Table 3. External Assessor Responses – Entrustable Profession Activities (EPAs) (n=23)**

| **EPAs Pre Items – Lung cancer scenario, breaking bad news** |  |  | **EPAs Post Items – Prostate cancer scenario, transitioning to end of life care** |  |  |
| --- | --- | --- | --- | --- | --- |
|  | **Mean** | **SD** |  | **Mean** | **SD** |
| Ascertain the patient's and/or the family's understanding of the medical situation and reason for the encounter | 2.28 | 0.33 | Convey complex or sensitive information regarding prognosis, plan of care, change in clinical status or uncertainty in a clear, compassionate, respectful, and accurate manner | 2.63 | 0.43 |
| Ascertain the patient's desire for amount and detail of information | 1.65 | 0.35 | Establish and implement ongoing plans for care which include but are not limited to referral to: a family physician; pain and symptom management/palliative care; home care/community services; psychological and spiritual support services | 2.50 | 0.43 |
| Engage the patient in shared decision-making regarding investigations and treatment options | 1.93 | 0.51 | Explore and confirm the patient's wishes regarding advance care planning | 2.28 | 0.60 |
| Identify, verify, and validate cues and respond with appropriate communication techniques to establish rapport | 1.87 | 0.63 | Manage the flow of challenging and emotionally charged patient encounters | 2.50 | 0.48 |
| Manage the flow of the encounter | 2.35 | 0.44 | Organize the handover of care to the most appropriate physician or health care professional | 1.91 | 0.49 |
| Provide effective explanations for the proposed goals of treatment and/or management | 2.13 | 0.53 | Reassess patient's priorities, information preferences and goals of care as the patient's clinical situation evolves | 2.52 | 0.53 |
| Share information in a compassionate, patient-centred and accurate manner | 2.15 | 0.57 | Respectfully discuss differences of opinion regarding discontinuation of direct anti-cancer therapy | 2.30 | 0.56 |
| Summarize and close the encounter effectively | 1.93 | 0.48 | Share concerns about patient's goals that may not be achievable and initiate discussion about addressing these concerns | 2.26 | 0.52 |
| Verify understanding of information conveyed | 1.67 | 0.51 |  |  |  |
| Breaking bad news EPA overall mean | 2.00 | 0.37 | Transitioning care EPA overall mean | 2.36 | 0.39 |

SD = Standard Deviation, Higher scores indicate higher proficiency (3-point scale)
